# Two-dimensional CsPbI_3_/CsPbBr_3_ vertical heterostructure: a potential photovoltaic absorber

**DOI:** 10.1038/s41598-023-48753-7

**Published:** 2023-12-06

**Authors:** Manushi J. Patel, Narayan N. Som, Sanjeev K. Gupta, P. N. Gajjar

**Affiliations:** 1 Department of Physics, University School of Sciences, Gujarat University, Ahmedabad, 380 009 Gujarat India; 2 Institute of High Pressure Physics, Polish Academy of Sciences, Sokolowska 29/37, 01-142 Warsaw, Poland; 3 Computational Materials and Nanoscience Group, Department of Physics and Electronics, St. Xavier’s College, Ahmedabad, 380 009 Gujarat India

**Keywords:** Surfaces, interfaces and thin films, Electronic devices

## Abstract

First-principles methods have been employed here to calculate structural, electronic and optical properties of CsPbI_3_ and CsPbBr_3_, in monolayer and heterostructure (HS) (PbI_2_-CsBr (HS1), CsI-CsBr (HS2), CsI-PbBr_2_ (HS3) and PbI_2_-PbBr_2_ (HS4)) configurations. Imaginary frequencies are absent in phonon dispersion curves of CsPbI_3_ and CsPbBr_3_ monolayers which depicts their dynamical stability. Values of interfacial binding energies signifies stability of our simulated heterostructures. The CsPbI_3_ monolayer, CsPbBr_3_ monolayer, HS1, HS2, HS3 and HS4 possess direct bandgap of 2.19 eV, 2.73 eV, 2.41 eV, 2.11 eV, 1.88 eV and 2.07 eV, respectively. In the HS3, interface interactions between its constituent monolayers causes substantial decrease in its resultant bandgap which suggests its solar cell applications. Static dielectric constants of all simulated heterostructures are higher when compared to those of pristine monolayers which demonstrates that these heterostructures possess low charge carrier recombination rate. In optical absorption plots of materials, the plot of HS3 displayed a red shift and depicted absorption of a substantial part of visible spectrum. Later on, via Shockley-Queisser limit we have calculated solar cell parameters of all the reported structures. The calculations showed that HS2, HS3 and HS4 showcased enhanced power conversion efficiency compared to CsPbI_3_ and CsPbBr_3_ monolayers when utilized as an absorber layer in solar cells.

## Introduction

Perovskites exhibiting various outstanding properties such as suitable bandgap, large absorption coefficient, good intrinsic carrier mobility etc. are one of the immensely studied class of materials^1–7^. Due to these extraordinary properties of perovskites a lot of efforts have been made for their applications in various optoelectronic devices such as LEDs, solar cells, and lasers^3,8–12^. Even though hybrid organic–inorganic metal halide perovskites e.g., MAPbI_3_, FAPbI_3_ etc. containing solar cells possess high power conversion efficiencies (PCEs), one of their major drawbacks is its instability in presence of heat and moisture^13,14^. Hence, here we have focused on completely inorganic metal halide perovskites i.e., cesium lead halides CsPbX_3_ (X = Br, I).

Recently, Yao et al. have created perovskite solar cell (PSC) based on 2D Ruddlesden-Popper (RP) CsPbI_3_ perovskite using 1-naphthylamine as a spacer which showed PCE of 16.62% and better stability^15^. A method was proposed by Choi et al. in which they added zwitterions to develop stable black *α*-phase CsPbI_3_ perovskites which when used in PSC exhibited PCE about 18.4%^16^. Also, theoretically it has been predicted that if TiO_2_ and Cu_2_FeSnS_4_ will be used as electron and hole transport materials, respectively, in CsPbBr_3_ based PSCs then resultant PCE up to 13.86% can possibly be achieved^17^. Xu et al.^18^ proposed that hole transport material free CsPbI_3_/CsSnI_3_ heterojunction-based PSC can be developed which possess PCE more than 19%. Theoretically, efficiencies as high as 28.75% has been yielded by using CsPbI_3_/FAPbI_3_ heterojunction as an absorber layer in PSCs^19^.

But nowadays, a shift in the focus is seen from 3D to 2D perovskites because of enhancement seen in the stability^20–22^. Now, one of the key problems with 2D monolayers is it is difficult to find a single monolayer possessing all the properties which are required to obtain good efficiency. Therefore, various techniques are being used to tune the properties of the monolayers like application of strain or electric field, doping etc.^21,23–27^. Similarly, one of the ways to improve properties of the monolayers is the formation of heterostructures (HSs) comprising of different monolayers^28–30^. In this HS configuration, individual monolayers show enhancement in their electronic and optical properties which improves the overall performance^28,31^. Previously, Singh et al. used density functional theory (DFT) to study MoS_2_/MAPbI_3_ HS and reported it to be one of the commendable materials for high efficiency PSCs^29^. Moreover, a HS of the type MoS_2_/BA_2_PbI_4_ 2D RP perovskite has been reported whose absorption analysis showed that the heterojunction present in this structure led to increase in its absorption intensity over a broad frequency range from which authors predicted its optoelectronic applications^30^. Also, a HS of the type CsPbBr_3_/CsPbI_3_ perovskite in orthorhombic phase has been investigated, the interface study of which has led to better understanding for its applications in solar cell devices^32^.

In the following paper, a comprehensive DFT-based study of CsPbI_3_ and CsPbBr_3_ monolayers has been done. Here we have reported structural, electronic, vibrational, and optical properties of CsPbI_3_ and CsPbBr_3_ monolayers. Also, effective masses of the charge carriers in these reported monolayers have been predicted. Later, calculations of solar cell parameters of these monolayers have been done via Shockley-Queisser (SQ) limit to study their photovoltaic applications^21,28,33^. We observed that CsPbI_3_ monolayer showed moderate efficiency when utilized as an absorber layer in photovoltaic cells but efficiency of CsPbBr_3_ monolayer for the similar application came out to be very low. According to our study, while going from bulk to their 2D counter parts perovskites tends to show increase in the bandgap^21,34,35^ which can be one of the vital reasons behind decrease in the efficiencies of the solar devices consisting of these monolayers as an absorber. Therefore, to tune the properties of these monolayers we have opted for simulating HSs (PbI_2_-CsBr (HS1), CsI-CsBr (HS2), CsI-PbBr_2_ (HS3) and PbI_2_-PbBr_2_ (HS4)) comprising of CsPbI_3_ monolayer and CsPbBr_3_ monolayer. We have also studied structural properties, electronic properties, formation energies, effective masses of charge carriers, optical properties, and solar cell applications of all possible HS configurations.

## Method: computational simulations

All the calculations reported in this research article were done within generalized gradient approximation (GGA) using Perdew-Burke-Ernzerhof (PBE) parametrization through SIESTA code^36–39^. The complete simulations were done by using norm-conserving pseudopotentials and double zeta polarization (DZP). For optimization and calculation of vibrational properties, respectively, 10 × 10 × 1 Monkhorst k-grid was applied. While a denser grid i.e., 31 × 31 × 1 Monkhorst k-grid was utilized to enhance accuracy of calculation of all electronic and optical properties of all the structures. For entire calculations energy cut-off was set to 300 Ry and was ensured that convergence was achieved. Here structural optimization was done until force on each atom of monolayers and heterostructures, respectively, was less than 10^–2^ eV/Å. The phonon spectra of the unit cells of crystal structures were calculated by considering density functional perturbation theory (DFPT) formalism^40^. Calculations of electronic and vibrational properties were done along Γ-M-X-R-Γ high symmetry directions. Here by using random phase approximation (RPA) all the optical properties were calculated^41^. These optical properties were simulated along optical vector (1.0, 1.0, 0.0) with the optical mesh of 31 × 31 ×  1.

## Results and discussion

### Crystal structure and stability

**Figure 1:**
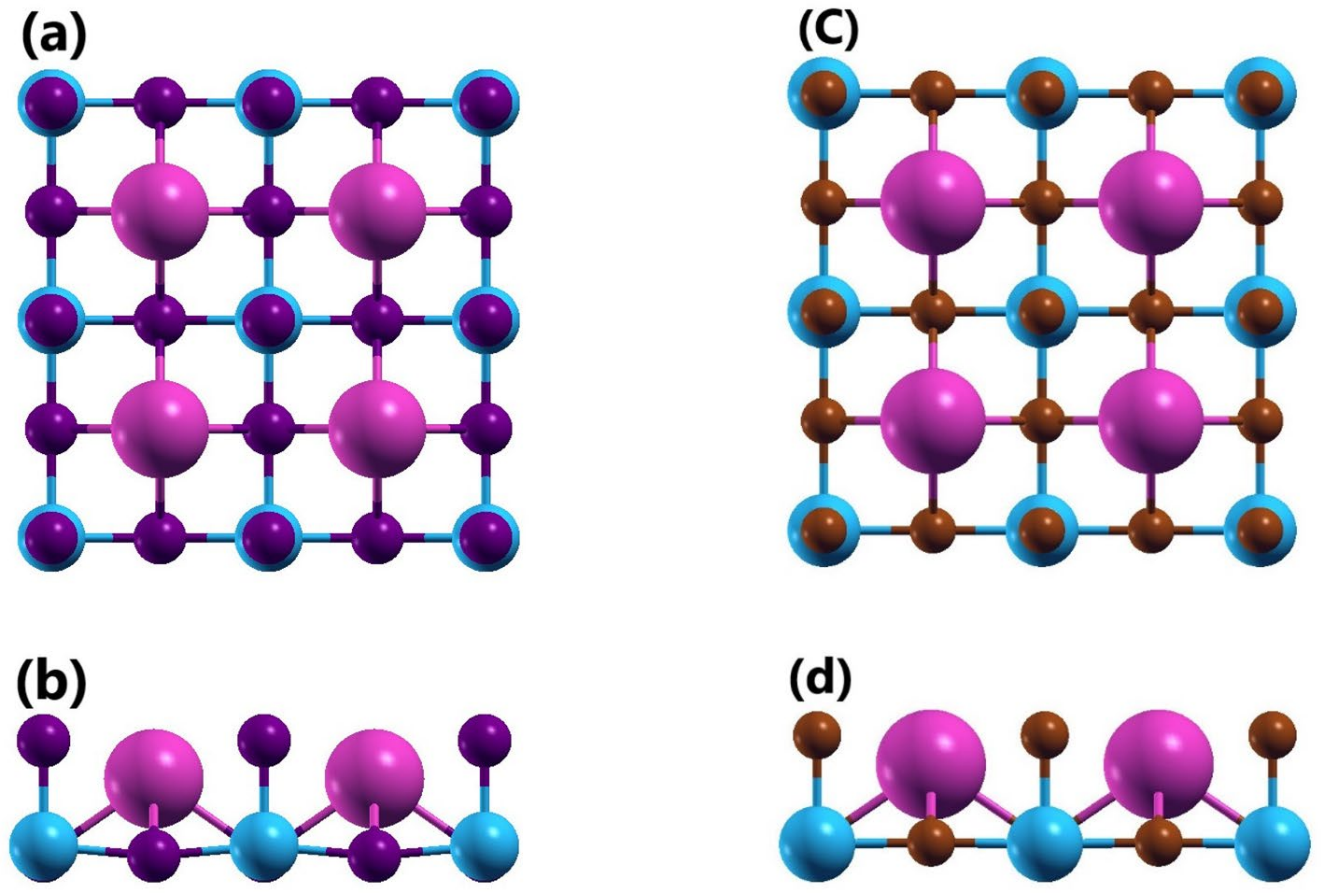
Crystal structure of CsPbI_3_ monolayer ((**a**) Top view and (**b**) Side view) and CsPbBr_3_ monolayer ((**c**) Top view and (**d**) Side view). Here pink, blue, purple and brown spheres represent Cs, Pb, I and Br atoms, respectively.

The CsPbI_3_ and CsPbBr_3_ monolayers have been formed by terminating their respective cubic phase bulk counterparts such that Cs-I/Br surface gets exposed on the top (Fig. S1,^34^). Here a 2 × 2 × 1 supercell consisting of 20 atoms have been simulated for monolayer formation. The crystal structures of these monolayers have been shown in Fig. 1a and c. The optimized lattice constants of CsPbI_3_ monolayer and CsPbBr_3_ monolayer simulated using GGA-PBE have been obtained to be about *a* = *b* = 12.86 Å and *a* = *b* = 12.03 Å, respectively. Also, optimization of CsPbI_3_ monolayer and CsPbBr_3_ monolayer have been carried out by introducing *DRSLL* dispersion correction method to take long range vdW interactions^42,43^ into consideration and their corresponding lattice parameters increased to *a* = *b* = 13.02 Å and *a* = *b* = 12.14 Å, respectively. In *z*-direction to avoid interactions distance of approximately 17 Å is present in all above-mentioned cases. The average bond lengths of Pb-I and Pb-Br present in CsPbI_3_ monolayer and CsPbBr_3_ monolayer optimized using GGA-PBE parametrization have been obtained about 3.15 Å and 2.94 Å, respectively. Here if vdW interactions are taken into consideration the average bond lengths of Pb-I and Pb-Br present in CsPbI_3_ monolayer and CsPbBr_3_ monolayer increases to 3.20 Å and 2.98 Å, respectively. Also, along with decrease in the size of halides present in the monolayer, the decrement is also seen in their corresponding Pb-X bond length^21^.

**Figure 2:**
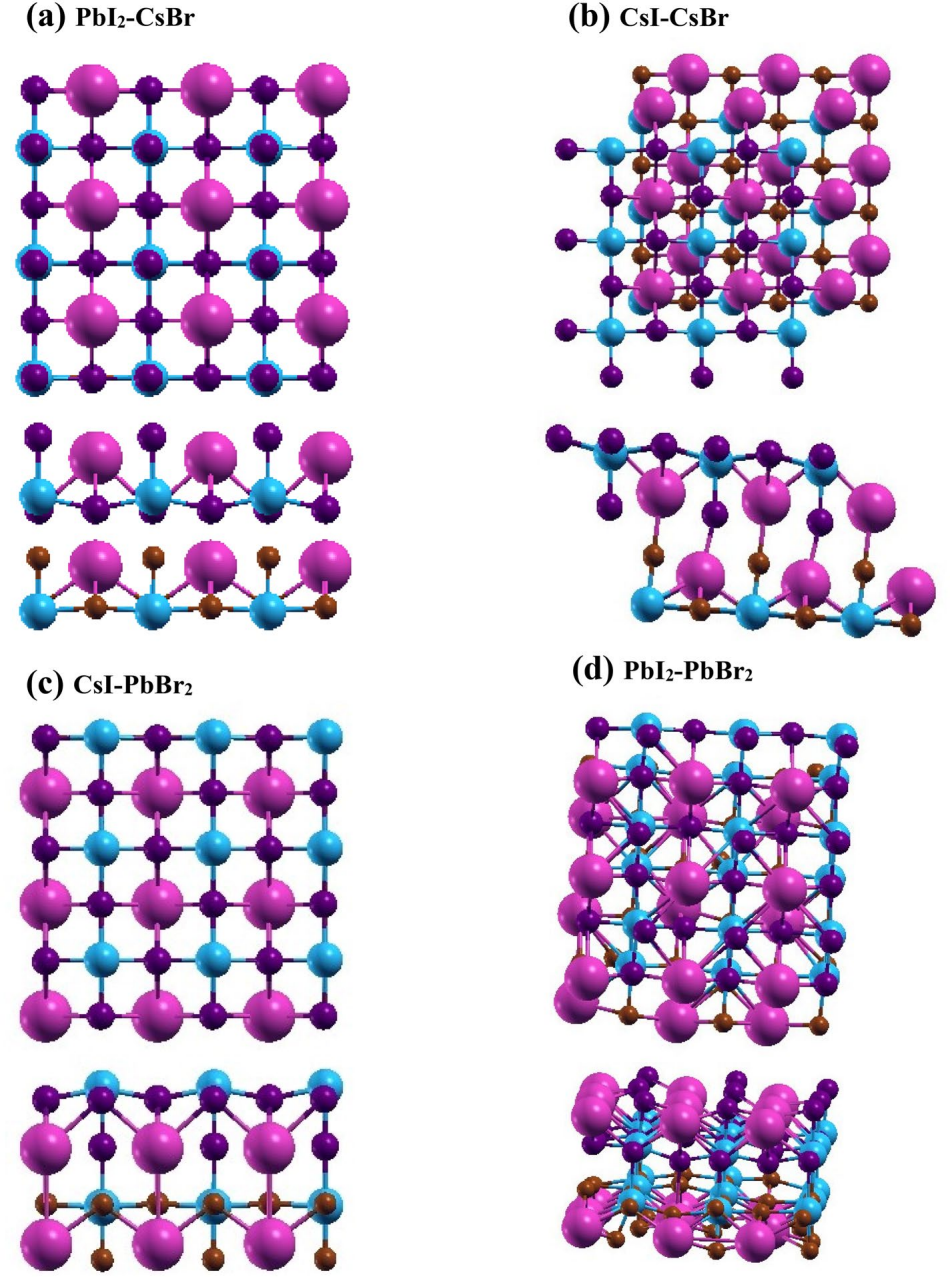
Illustration of top view and side view of crystal structure of (**a**) HS1, (**b**) HS2, (**c**) HS3 and (**d**) HS4. Here pink, blue, purple and brown spheres represent Cs, Pb, I and Br atoms, respectively.

Now, it is clear from Fig. 1 that CsPbI_3_ monolayer and CsPbBr_3_ monolayer possess two types of interfaces which are Cs-I/Br and Pb-I/Br. Based on these interfaces four different types of heterostructures have been formed which are PbI_2_-CsBr (HS1), CsI-CsBr (HS2), CsI-PbBr_2_ (HS3) and PbI_2_-PbBr_2_ (HS4) as shown in Fig. 2. Here the supercells of heterostructures developed consists of 90 atoms. The lattice parameters of HS1 are *a* = *b* = 18.78 Å (α = 90.37°, β = 90.39°, γ = 90°), HS2 are *a* = *b* = 18.78 Å (α = 110.65°, β = 110.53°, γ = 89.84°), HS3 are *a* = *b* = 18.80 Å (α = 89.31°, β = 89.30°, γ = 90.01°) and of HS4 are *a* = 18.89 Å, *b* = 19.29 Å (α = 63.37°, β = 87.47°, γ = 90.25°). In the heterostructures, to avoid interactions along *z*-direction, distances of approximately 25 Å, 36 Å, 29 Å and 25 Å are present in optimized HS1, HS2, HS3 and HS4 respectively. The interlayer distances between CsPbI_3_ monolayer and CsPbBr_3_ monolayer present in optimized HS1, HS2, HS3 and HS4 are 3.33 Å, 3.77 Å, 3.37 Å and 3.45 Å respectively. Here in the heterostructures both the monolayers present experiences strain due to the presence of lattice mismatch. The CsPbI_3_ monolayer experiences − 4% compressive strain while CsPbBr_3_ experiences + 3% tensile strain in the proposed heterostructures. Also, the influences of these strains on individual monolayers have been studied. Crystal structures of strained monolayers shown in Fig. S2, depicts that at the given strain none of the monolayers gets distorted. Also, it is clear from their negative cohesive energies (Table S2) that both the strained monolayers are relatively stable.

**Figure 3:**
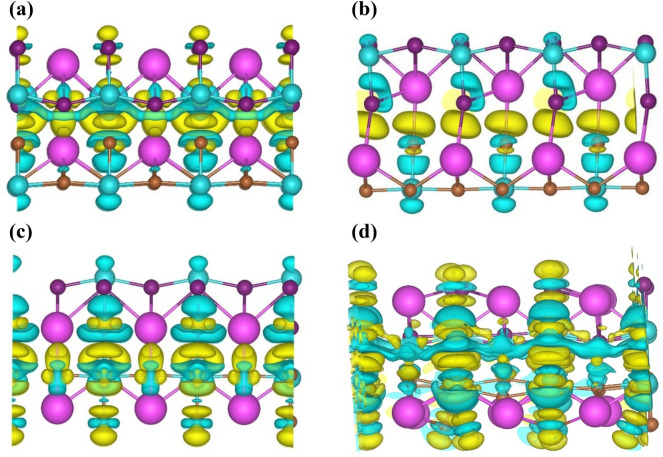
Charge density difference plots of (**a**) HS1, (**b**) HS2, (**c**) HS3 and (**d**) HS4. Here we have set isosurfaces at 0.0021 e/Å^3^ for all the heterostructures. Also, pink, blue, purple and brown spheres represent Cs, Pb, I and Br atoms, respectively. The yellow region represents charge accumulation and blue region represents charge depletion.

In order to investigate how charge accumulation and depletion regions are formed at the interface of CsPbI_3_ monolayer and CsPbBr_3_ monolayer in the heterostructures we have plotted the charge density difference plots of these heterostructures. The formula used to calculate charge density difference plots is as follows,

1 $$\Delta \rho = \rho_{HS} - \rho_{{CsPbI_{3} }} - \rho_{{CsPbBr_{3} }}$$

where in Eq. (1), $\rho_{HS}$ represents charge density of HS; $\rho_{{CsPbI_{3} }}$ and $\rho_{{CsPbBr_{3} }}$ represents charge density of isolated CsPbI_3_ monolayer and CsPbBr_3_ monolayer, respectively. These charge density difference plots of all the heterostructures have been shown in Fig. 3. It can be noticed from Fig. 3a, that a continuous charge depletion region is formed around Pb atoms of CsPbI_3_ monolayer at interface in HS1 while the intermittent charge accumulation takes place in the regions near to CsPbBr_3_ monolayer of HS1. In HS2 (Fig. 3b), maximum charge accumulation takes place at interface of the constituent monolayers. While in case of HS3 (Fig. 3c), at interface, charge accumulation occurs around Cs atoms of CsPbI_3_ monolayer and charge depletion occurs around Pb atoms of CsPbBr_3_ monolayer. Similar to HS1, according to Fig. 3d, HS4 also possess a continuous charge depletion region near CsPbI_3_ monolayer.

**Figure 4:**
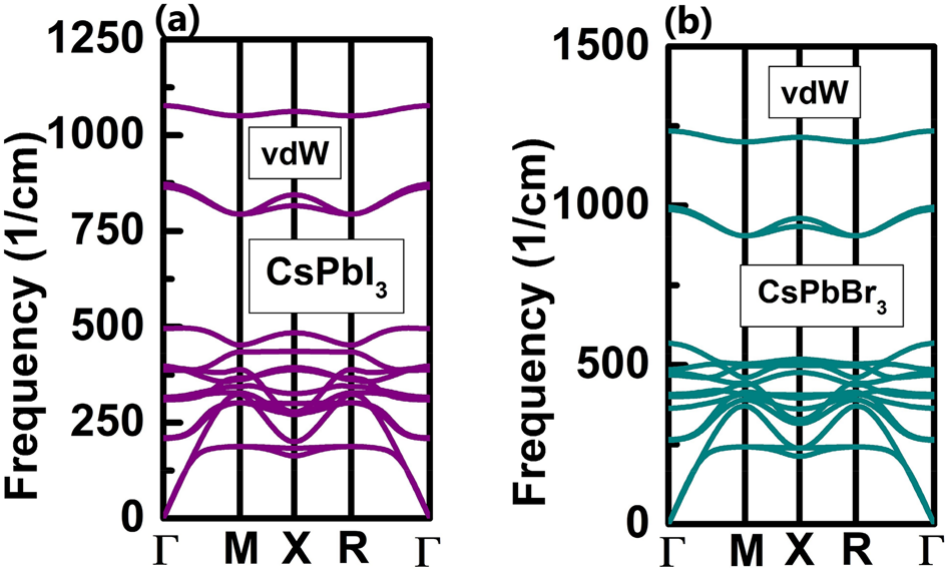
Phonon dispersion curves of unit cells of (**a**) CsPbI_3_ monolayer and (**b**) CsPbBr_3_ monolayer obtained by taking vdW interactions into consideration.

Firstly, to confirm dynamical stability of our proposed monolayers the phonon dispersion curves of the unit cell of our respective monolayers have been calculated. The phonons of unit cell of CsPbI_3_ monolayer and CsPbBr_3_ monolayer, respectively, have been calculated using both GGA-PBE parametrization (Fig. S3) as well as by including vdW interactions (Fig. 4). Here imaginary frequencies are absent in their respective phonon dispersion curves which shows stability of our reported monolayers.

As unit cells of the monolayers consists of 5 atoms, it can be noticed that their phonon dispersion curves possess 15 branches. From these 15 branches, there are 3 acoustical branches and 12 optical branches. Highest phonon frequencies obtained are 1169.45/cm and 1316.26/cm for CsPbI_3_ monolayer and CsPbBr_3_ monolayer, respectively, in their phonon dispersion curves. Here along with decrease in the ionic radii of halides in these inorganic monolayers an increase has been observed in their corresponding highest phonon frequencies^21^. Similar nature of change in highest phonon frequencies have also been observed in case of bulk CsPbX_3_ (X = Cl, Br, I)^34^. Also, highest phonon frequencies obtained for these monolayers by taking vdW interactions into consideration are 1076.80/cm and 1234.56/cm for CsPbI_3_ monolayer and CsPbBr_3_ monolayer, respectively. Hence, it can be observed here that even though nature of phonon dispersion curves remains same but due to consideration of vdW interactions there is decrease seen in highest phonon frequencies for both the monolayers.

In order to study magnitude of interfacial adhesion present in our reported heterostructures their corresponding interfacial binding energies have been calculated using equation given below^30,44^

2 $$\Delta {\text{E }} = \frac{{E_{HS} - E_{{CsPbI_{3} }} - E_{{CsPbBr_{3} }} }}{S}$$

where ΔE represents interfacial binding energy of corresponding heterostructure; $E_{HS}$, $E_{{CsPbI_{3} }}$ and $E_{{CsPbBr_{3} }}$ signifies total energies of the heterostructures, monolayer CsPbI_3_ and monolayer CsPbBr_3_, respectively; *S* represents area of the interface. The interfacial binding energies calculated for HS1 (PbI_2_-CsBr), HS2 (CsI-CsBr), HS3 (CsI-PbBr_2_) and HS4 (PbI_2_-PbBr_2_) using Eq. (2) have been obtained about − 23.58 meV/Å^2^, − 16.81 meV/Å^2^, − 17.18 meV/Å^2^ and − 23.56 meV/Å^2^, respectively. Here the values of binding energies suggest stability of all our simulated heterostructures.

### Electronic properties

**Figure 5:**
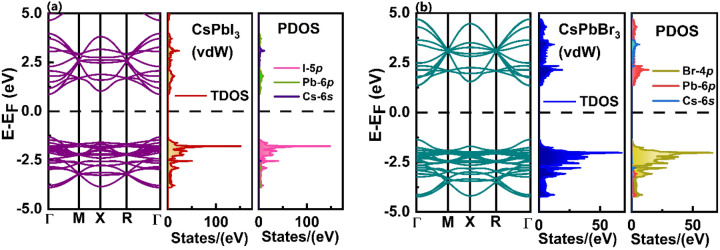
Band structure, TDOS and PDOS of (**a**) CsPbI_3_ monolayer and (**b**) CsPbBr_3_ monolayer obtained by taking into consideration vdW interactions. Here electronic properties calculated is of 2 × 2 × 1 monolayer consisting of 20 atoms.

The study of electronic properties of any structures is quite significant to predict their potential applications. Therefore, detailed analysis of electronic properties of CsPbI_3_ monolayer, CsPbBr_3_ monolayer as well as the heterostructures (HS1, HS2, HS3 and HS4) has been done. The band structure, total density of states (TDOS) and partial density of states (PDOS) of CsPbI_3_ monolayer and CsPbBr_3_ monolayer have been depicted in Fig. 5 and S4﻿. Both these monolayers are direct bandgap semiconductors and their gaps have been obtained at Γ-points. Monolayer CsPbI_3_ and monolayer CsPbBr_3_ possess bandgap of 2.19 eV and 2.73 eV, respectively, obtained by taking into consideration vdW interactions. The increase in bandgaps of these inorganic perovskite monolayers is seen while going from bulk to 2D which is due to quantum confinement effect^34,45,46^. Here it can be noticed that when the internal vdW interactions present in these monolayers have been taken into consideration they tend to show slight increase in their bandgaps (Table S3). Also, major peaks in TDOS and PDOS of CsPbI_3_ monolayer and CsPbBr_3_ monolayer occurs at − 1.78 eV and − 2.02 eV, respectively, in valence band region which arises due to their corresponding I-5*p* and Br-4*p* orbitals, respectively. It can be noticed that the conduction band edges are determined by Pb-6*p* orbitals, while the valence band edges are determined by I-5*p* and Br-4*p* orbitals for CsPbI_3_ monolayer and CsPbBr_3_ monolayer, respectively. There is no major contribution from Cs-6*s* orbital at the band edges of the monolayers. Similar kind of behavior has been seen earlier in case of bulk CsPbX_3_ (X = I, Br)^34^.

**Figure 6:**
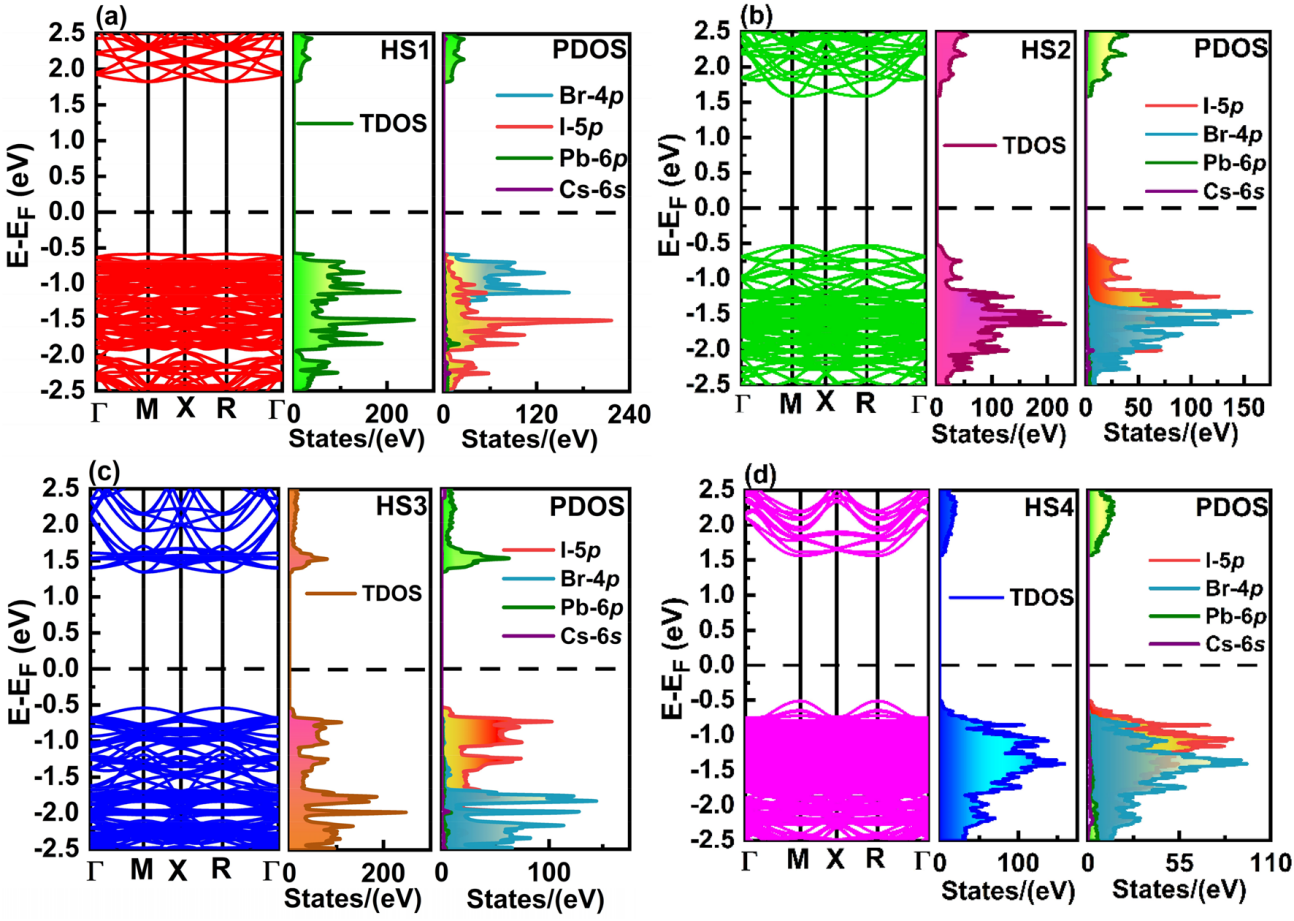
Band structure, TDOS and PDOS of heterostructures (**a**) HS1, (**b**) HS2, (**c**) HS3 and (**d**) HS4.

From Fig. 6, one can conclude that all the heterostructures are direct bandgap semiconductors with energy gaps obtained at M- and R- points. Here depending on the interactions taking place between the constituent monolayers of the corresponding heterostructure they possess different energy gaps. The resultant energy gaps obtained in HS1 (PbI_2_-CsBr), HS2 (CsI-CsBr), HS3 (CsI-PbBr_2_) and HS4 (PbI_2_-PbBr_2_) have been about 2.41 eV, 2.11 eV, 1.88 eV and 2.07 eV, respectively. Here PDOS of the HSs show that the Cs-6* s* orbitals of its constituent monolayers have no significant role in determining its band edges. From the TDOS and PDOS of the HSs one can conclude that maximum contribution in determining its valence band region have been of I-5*p* and Br-4*p* orbitals. While there has been significant influence of Pb-6*p* orbitals of constituent monolayers of HS in determining its conduction band region.

It has been known that materials which exhibits charge carriers of low effective masses tends to show better charge transport properties which leads to their application in various optoelectronic devices. We have also computed effective masses of electrons $\left( {m_{e}^{*} } \right)$ and holes $\left( {m_{h}^{*} } \right)$ for all the monolayers and HSs using method of parabolic curve fitting around the conduction band minima and valence band maxima occurring in their band structures. The formula used for the calculations of effective masses is as follows:

3 $$m^{*} = \frac{{\hbar^{2} }}{{\frac{{d^{2} E}}{{dk^{2} }}}}$$

The effective masses of electrons and holes for the reported structures have been tabulated in the Table 1.

**Table 1:** Effective masses of charge carriers calculated for CsPbI_3_ monolayer, CsPbBr_3_ monolayer, HS1, HS2, HS3 and HS4.

Structures		$m_{e}^{*} /m_{o}$	$m_{h}^{*} /m_{o}$
CsPbI_3_ monolayer		0.06	0.06
CsPbBr_3_ monolayer		0.06	0.07
HS1 (PbI_2_-CsBr)	From Γ to M point	0.30	0.25
	From X to R point	0.38	0.30
HS2 (CsI-CsBr)	From Γ to M point	0.19	0.48
	From X to R point	0.28	0.68
HS3 (CsI-PbBr_2_)	From Γ to M point	0.08	0.26
	From X to R point	0.10	0.30
HS4 (PbI_2_-PbBr_2_)	From Γ to M point	0.14	0.07
	From X to R point	0.15	0.08

Here from all the studied heterostructures it can be concluded that HS1 possess highest effective masses of electrons while HS3 exhibits lowest electron effective masses. This shows that transport of electrons is most facilitated in HS3 compared to that of other HSs. Similarly, effective masses of holes are lowest in HS4 and highest in HS2. While the effective masses of holes are almost similar in HS1 and HS3. Also, it has been studied that the effective masses of charge carriers in our reported heterostructures are quite impressive when compared to that of many previously reported perovskites and perovskite HSs^30,47,48^. This shows that our stated HS1 (PbI_2_-CsBr), HS2 (CsI-CsBr), HS3 (CsI-PbBr_2_) and HS4 (PbI_2_-PbBr_2_) are all suitable candidates to be used as a solar cell absorber.

### Optical properties

The complex dielectric function (ε(ω)) can be given as,

4 $$\varepsilon \left( \omega \right) \, = \, \varepsilon_{{\text{r}}} \left( \omega \right) \, + i\varepsilon_{{\text{i}}} \left( \omega \right)$$

**Figure 7:**
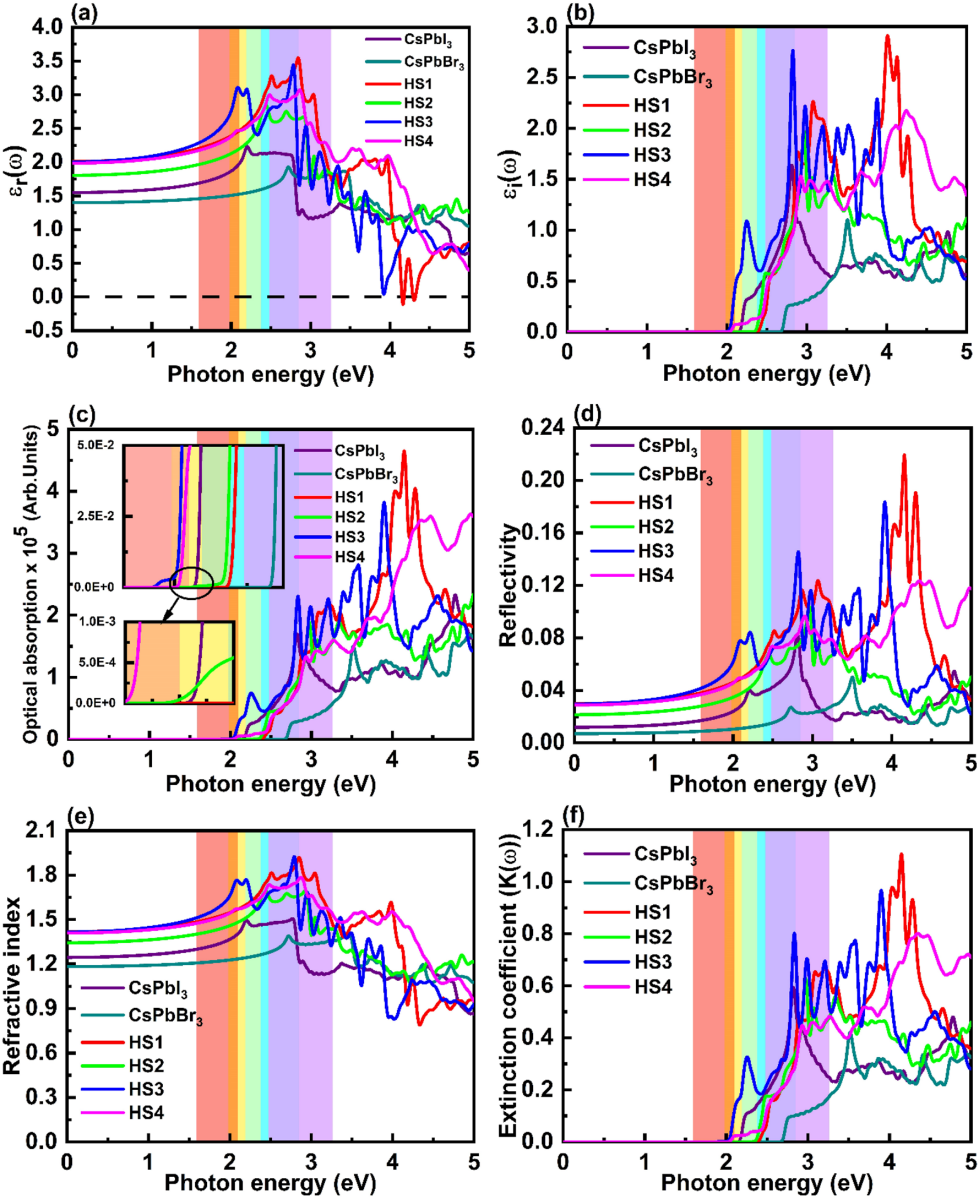
Plots of change in (**a**) real and (**b**) imaginary parts of dielectric constants, (**c**) optical absorption (here inset shows first peak edges of all the structures), (**d**) reflectivity, (**e**) refractive indices and (**f**) extinction coefficient along with increase in incoming photon energy for CsPbI_3_ monolayer, CsPbBr_3_ monolayer, HS1, HS2, HS3 and HS4, respectively. Here the heterostructures possess 3 × 3 × 1 constituent monolayer therefore in order to show comparison we have plotted optical properties of optimized 3 × 3 × 1 pristine CsPbI_3_ and CsPbBr_3_ monolayers.

Here in Eq. (4), ε_r_(ω) and ε_i_(ω) indicates real and imaginary parts of a dielectric constant, respectively. It can be noted from Fig. 7a, that HS1 possess negative values of ε_r_(ω) at photon energies 4.17 eV as well as 4.31 eV. These negative values of ε_r_(ω) depicts that HS1 shows metallic character corresponding to that incident photon energy^49^. Also, it can be observed that the values of static dielectric constant i.e., ε_r_(0) is smaller for the monolayers while its value enhances in the heterostructures. Through Clausius–Mossotti relation, using ε_r_(ω), we can get some evidence regarding electronic polarizability of the proposed materials^50^. Here as mentioned above ε_r_(0) is lower in case of monolayers thus showing lower polarizability in them compared to heterostructures. Also, materials possessing high dielectric constants tends to show low charge carrier recombination rate^51^. Hence it can be said here that while going from monolayers to the heterostructures there will be reduction observed in carrier recombination rate thus supporting HS’s application in the solar cells. In visible region, the plots of ε_r_(ω) shows peaks at 2.84 eV (blue region), 2.44 eV (cyan region), 2.78 eV (blue region) and 2.86 eV (violet region) in case of HS1, HS2, HS3 and HS4, respectively. The plots of ε_i_(ω) as a function of incoming photon energy for all the structures have been shown in Fig. 7b. For CsPbI_3_ monolayer, HS2 and HS3, graphs of ε_i_(ω) shows highest peaks at 2.81 eV (blue region), 2.96 eV (violet region) and 2.82 eV (blue region), respectively, in the visible region. In case of CsPbBr_3_ monolayer, HS1 and HS4 there are peaks in the plots of ε_i_(ω) at 3.51 eV, 4.01 eV and 4.25 eV, respectively, obtained in the ultraviolet region.

Figure 7c shows nature of change in optical absorption of the materials depending on incoming photon energy. It can be noticed that due to the nature of interactions between two monolayers in HS3 a red shift has been observed in its absorption edge (visible (red) region) and it covers significant part of the visible spectrum. Hence it can be said that HS3 is most appropriate candidate for solar cell applications. Also, highest peaks in absorption coefficient plots of all the proposed structures have been obtained in ultraviolet region which depicts that in corresponding region these materials absorb more photons. Figure 7d shows that the static reflectivity i.e., reflectivity at zero photon energy is lowest in case of CsPbBr_3_ monolayer. The values of static reflectivity for CsPbI_3_ monolayer, CsPbBr_3_ monolayer, HS1, HS2, HS3 and HS4 have been obtained about 1.19%, 0.70%, 2.99%, 2.15%, 2.96% and 2.88%, respectively. The maxima peak in reflectivity plots for CsPbI_3_ monolayer and CsPbBr_3_ monolayer have been obtained at 2.81 eV and 3.50 eV, respectively, lying in visible (blue region) and near ultraviolet regions, respectively. While in heterostructures, the HS2 shows highest peak in reflectivity plot in visible (violet) region where as the remaining HSs show highest peak in ultraviolet region. Also, peaks occur in reflectivity plot of HS1 in mid ultraviolet region which is as discussed above due to its metallic characteristics around that region. Figure 7e shows occurrence of changes in refractive indices plots depending on increase in incoming photon energy for all the reported monolayers and HSs. The maximum values of refractive indices for CsPbI_3_ monolayer, HS1, HS2, HS3 and HS4 are 1.50 (blue region), 1.92 (blue region), 1.69 (cyan and violet region), 1.93 (blue region) and 1.79 (violet region), respectively, all of which have been located in visible region. While in case of CsPbBr_3_ monolayer maximum value of refractive index i.e., 1.40 have been obtained in visible (blue region) as well as near ultraviolet region. Here like reflectivity, the static refractive indices i.e., refractive index at zero photon energy have been higher in case of heterostructures compared to that of pristine monolayers. The extinction coefficients (K(ω)) of the reported materials shown in Fig. 7f have been calculated using following equations^21^,

5 $${\text{K}}(\omega ) = \sqrt {\frac{{\left| {\varepsilon \left( \omega \right)} \right| - \varepsilon_{r} \left( \omega \right)}}{2}} ,\;\left| {\varepsilon \left( \omega \right)} \right| = \sqrt {(\varepsilon_{r} \left( \omega \right))^{2} + (\varepsilon_{i} \left( \omega \right))^{2} }$$

In extinction coefficient plots, peaks are located at 2.82 eV, 3.52 eV, 4.15 eV, 2.96 eV, 3.89 eV and 4.35 eV for CsPbI_3_ monolayer, CsPbBr_3_ monolayer, HS1, HS2, HS3 and HS4 respectively, hence photons with these energies will be absorbed more by the materials and they will pierce least into the materials^21,49^. The values of extinction coefficients at the energy at which maxima peaks have been located are 0.60, 0.42, 1.11, 0.63, 0.97 and 0.81 for CsPbI_3_ monolayer, CsPbBr_3_ monolayer, HS1, HS2, HS3 and HS4, respectively. Now, from the energy at which extinction coefficient plot shows maxima decay length have been calculated through formula given below^50^,

6 $${\text{Decay length }} = \frac{c}{{\omega \times \left( {K\left( \omega \right)} \right)}}$$

Here $c$ denotes speed of light and $\omega$ shows angular frequency corresponding to energy at which maxima is obtained in Fig. 7f. The decay lengths of CsPbI_3_ monolayer, CsPbBr_3_ monolayer, HS1, HS2, HS3 and HS4 have been obtained about 1167.64 Å, 1336.34 Å, 428.88 Å, 1059.44 Å, 523.59 Å and 560.71 Å, respectively. Hence, one can conclude that while going from monolayers to heterostructures a decrement has been observed in decay lengths of the materials.

### Solar cell parameters

Here solar cell parameters like short circuit current density (*J*_*sc*_), open-circuit voltage (*ʋ*_*oc*_) and power conversion efficiency (*ƞ*) have been calculated for all the reported structures using SQ limit^21,28,33^. The *ƞ* is calculated using the equation given below,

7 $$\eta = \frac{{J_{sc} \times \upsilon_{oc } \times FF}}{{P_{in} }}$$

where in Eq. (7), $P_{in}$ represents total incident solar energy density which is 1000 W/m^2^^52^. The formula to find *J*_*sc*_ is as follows,

8 $$J_{sc} = \mathop \smallint \limits_{0}^{\infty } eA\left( E \right)I_{sun} \left( E \right)dE$$

Here $A\left( E \right)$ in Eq. (8) shows absorptivity which is a Heaviside step function in SQ limit^53^. Here photon flux density i.e., $I_{sun} \left( E \right)$ is taken from AM1.5G spectrum^54^ and $e$ represents elementary charge. In case of monolayers formulae to calculate total current density (*J*) and reverse saturation current density (*J*_*o*_) are shown^21,34^,

9 $$J \, = \, J_{sc} {-} \, J_{o} \left( {e^{{\frac{eV}{{kT}}}} - 1} \right)$$

10 $$J_{o} = \mathop \smallint \limits_{0}^{\infty } e\pi A\left( E \right)I_{bb} \left( {E,T} \right)dE$$

In Eq. (9),* V* present shows potential across absorber layer, *k* represents Boltzmann’s constant and *T* = 300 K which is temperature of considered photovoltaic cell. Here in Eq. (10), *I*_*bb*_*(E,T)* represents black body spectrum at temperature *T*^53^. The open-circuit voltage (*ʋ*_*oc*_) for monolayers have been calculated using equation given below^28^,

11 $$\upsilon_{oc} = \frac{kT}{q}{\text{ln}}\left( {\frac{{J_{sc} }}{{J_{o} }} + 1} \right)$$

For heterostructures, *ʋ*_*oc*_ is modified according to below formula^28^,

12 $$\upsilon_{oc} = \left( {E_{g} {-} \, CBO \, {-} \, 0.3} \right)$$

Here in Eq. (12), *E*_*g*_ represents donor band gap, *CBO* shows conduction band off set and 0.3 eV is loss because of energy conversion kinetics. Here for all the cases $FF$ is considered 0.65^28^.

The calculated values of *J*_*sc*_ for CsPbI_3_ monolayer, CsPbBr_3_ monolayer, HS1, HS2, HS3 and HS4 are 106.64 A/m^2^, 36.11 A/m^2^, 72.44 A/m^2^, 121.34 A/m^2^, 175.62 A/m^2^ and 129.23 A/m^2^, respectively. Here it can be noted that value of *J*_*sc*_ is minimum for CsPbBr_3_ monolayer while its maximum value has been obtained in HS3. Therefore, it can be concluded here that as the bandgap of the materials increases their corresponding values of *J*_*sc*_ decreases. Here from Table 2 which depicts calculated efficiencies of all monolayers and HSs, it can be noticed that with suitable bandgap tuning the efficiency of the solar cells can be increased up to 23.40% which has been obtained in case of HS3 (CsI-PbBr_2_).

**Table 2:** Efficiencies of CsPbI_3_ monolayer, CsPbBr_3_ monolayer, HS1, HS2, HS3 and HS4.

Structure	Efficiency (*ƞ*)
CsPbI_3_ monolayer	13.10%
CsPbBr_3_ monolayer	5.61%
HS1 (PbI_2_-CsBr)	12.15%
HS2 (CsI-CsBr)	18.00%
HS3 (CsI-PbBr_2_)	23.40%
HS4 (PbI_2_-PbBr_2_)	18.84%

## Conclusions

Therefore, a detailed DFT based investigation of CsPbI_3_ monolayer and CsPbBr_3_ monolayer showed that they are direct bandgap semiconductor possessing energy gap of 2.19 eV and 2.73 eV, respectively. These monolayers being wide bandgap semiconductors showcased average PCE when utilized as absorber layer in solar cells. Hence, we opted the method of tuning the bandgaps of these monolayers by forming their HSs. We managed to simulate four different types of heterostructures i.e., PbI_2_-CsBr (HS1), CsI-CsBr (HS2), CsI-PbBr_2_ (HS3) and PbI_2_-PbBr_2_ (HS4) from these perovskite monolayers. Later, after studying electronic properties of these HS1, HS2, HS3 and HS4 we found that they are all direct bandgap semiconductors depicting energy gap of 2.41 eV, 2.11 eV, 1.88 eV and 2.07 eV, respectively. A substantial decrease in energy gap is observed in case of HS3 due to interaction between two constituent monolayers. Also, analysis of optical properties of HS3 further promotes its use as absorption layer in PSCs. Finally, we then considered calculating solar cell parameters of HS3 (CsI-PbBr_2_) which showed *J*_*sc*_ and PCE about 175.62 A/m^2^ and 23.40%, respectively. Hence it can be concluded here that the method of forming heterostructures from the perovskite monolayers is one of the effective ways to develop high efficiency PSCs.

### Supplementary Information

Supplementary Information.

## Data Availability

The datasets generated and/or analysed during the current study are not publicly available due to privacy or other restrictions. However, it may be made available from the corresponding author on reasonable request.
