# Supplementary material for: Two-dimensional CsPbI3/CsPbBr3 vertical heterostructure: a potential photovoltaic absorber
Source: Sci Rep. 2023 Dec 6;13:21551. doi: 10.1038/s41598-023-48753-7 (PMC10700334; doi:10.1038/s41598-023-48753-7)
Supplement: Supplementary file 1 — Supplementary Information. [file 41598_2023_48753_MOESM1_ESM.docx]

**Supporting Information**

**Two-dimensional CsPbI_3_/CsPbBr_3_ vertical heterostructure: A potential photovoltaic absorber**

Manushi J. Patel ^a^, Narayan N. Som ^b^, Sanjeev K. Gupta ^c, *^ and P. N. Gajjar ^a, *^

^a^ *Department of Physics, University School of Sciences, Gujarat University, Ahmedabad 380 009, Gujarat, India*

^b^ *Institute of High Pressure Physics, Polish Academy of Sciences, Sokolowska 29/37, 01-142, Warsaw, Poland*

^c^ *Computational Materials and Nanoscience Group, Department of Physics and Electronics, St. Xavier’s College, Ahmedabad 380 009, Gujarat, India*

**Cubic phase bulk CsPbX_3_ (X = I, Br)**

Here optimization of cubic phase bulk CsPbI_3_ and CsPbBr_3_ has been carried out by taking into consideration the vdW interactions.

| **(a)**  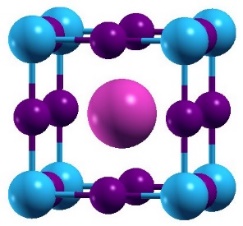 | 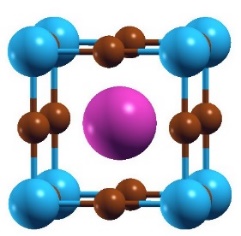 **(b)** |
| --- | --- |
| 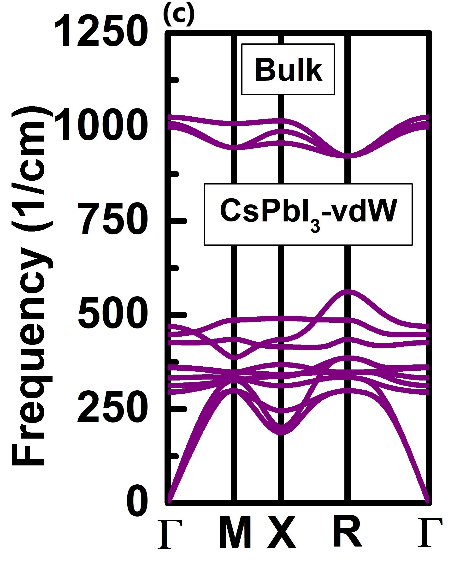 | 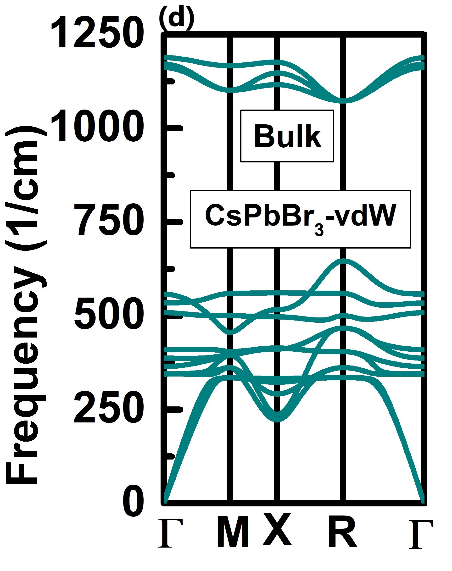 |

**Fig. S1.** Crystal structure of cubic phase bulk (a) CsPbI_3_ and (b) CsPbBr_3_. Here pink, blue, purple and brown spheres represent Cs, Pb, I and Br atoms, respectively. Phonon dispersion curves of cubic phase bulk (c) CsPbI_3_ and (d) CsPbBr_3_.

TABLE S1: Lattice parameters of cubic phase bulk CsPbI_3_ and CsPbBr_3_ obtained by considering vdW interactions.

| Structure | Lattice parameters |
| --- | --- |
| CsPbI_3_ | *a = b = c =* 6.49 Å [1] |
| CsPbBr_3_ | *a = b = c =* 6.02 Å [1] |

**Strained monolayers present in heterostructures**

| 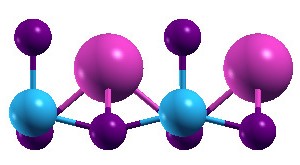 **(a)**  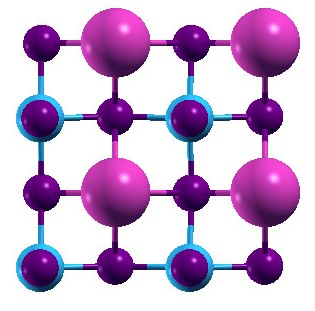 | **(b)**  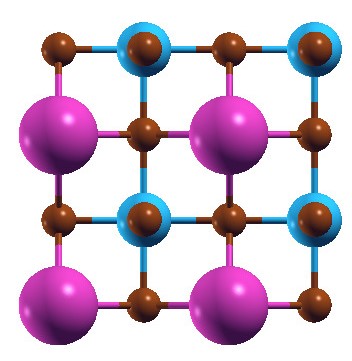  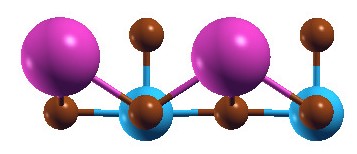 |
| --- | --- |

**Fig. S2.** (a) Top view and side view of crystal structure of CsPbI_3_ monolayer at – 4% compressive strain. (b) Top view and side view of crystal structure of CsPbBr_3_ monolayer at + 3% tensile strain. Here pink, blue, purple and brown spheres represent Cs, Pb, I and Br atoms, respectively.

TABLE S2: Cohesive energies of strained monolayers

| Structure | Cohesive energy (eV/atom) |
| --- | --- |
| CsPbI_3_ (- 4% compressive strain) | -3.98 |
| CsPbBr_3_ (+ 3% tensile strain) | -4.28 |

**Phonons of CsPbI_3_ monolayer and CsPbBr_3_ monolayer obtained using GGA-PBE parametrization**

| 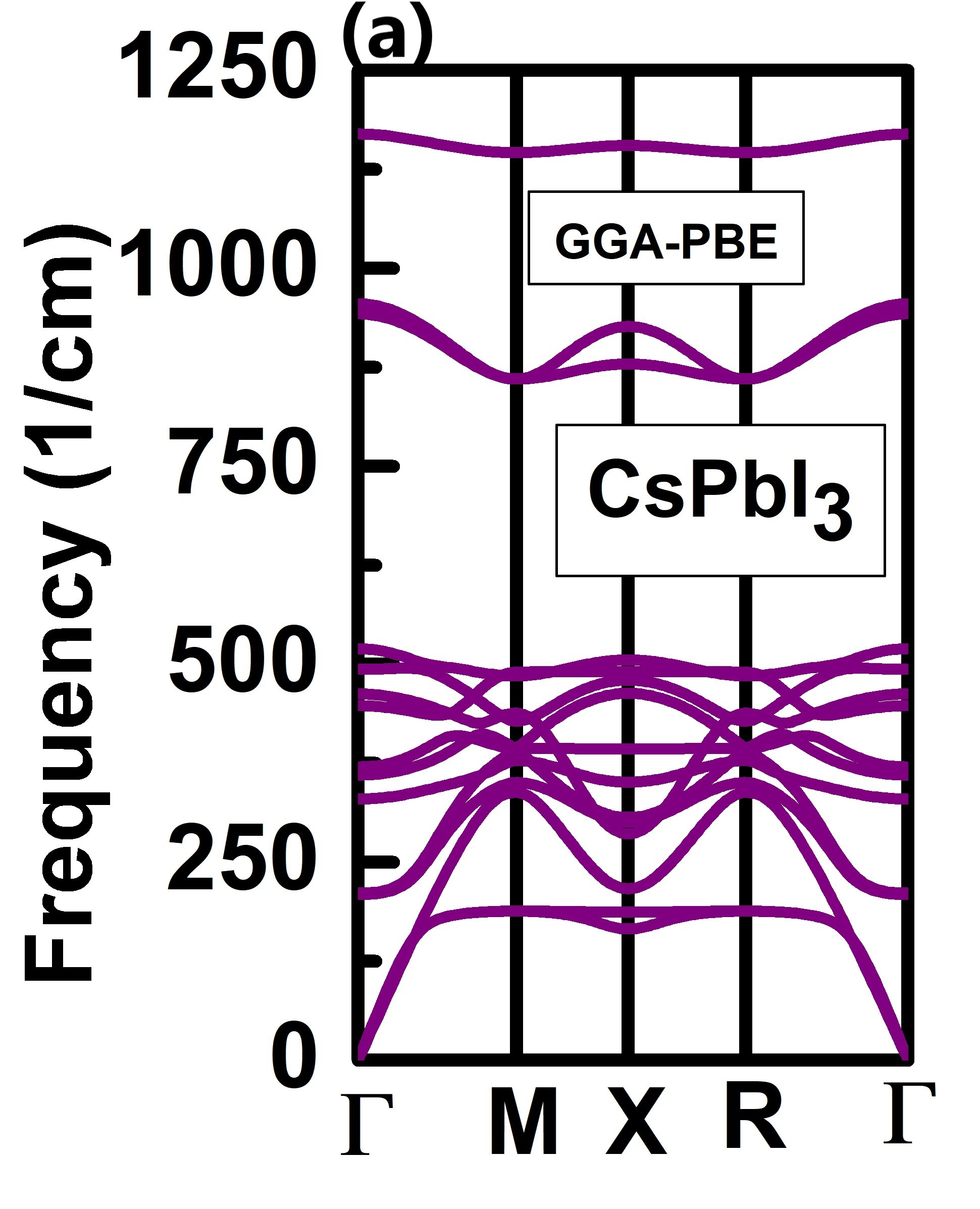 | 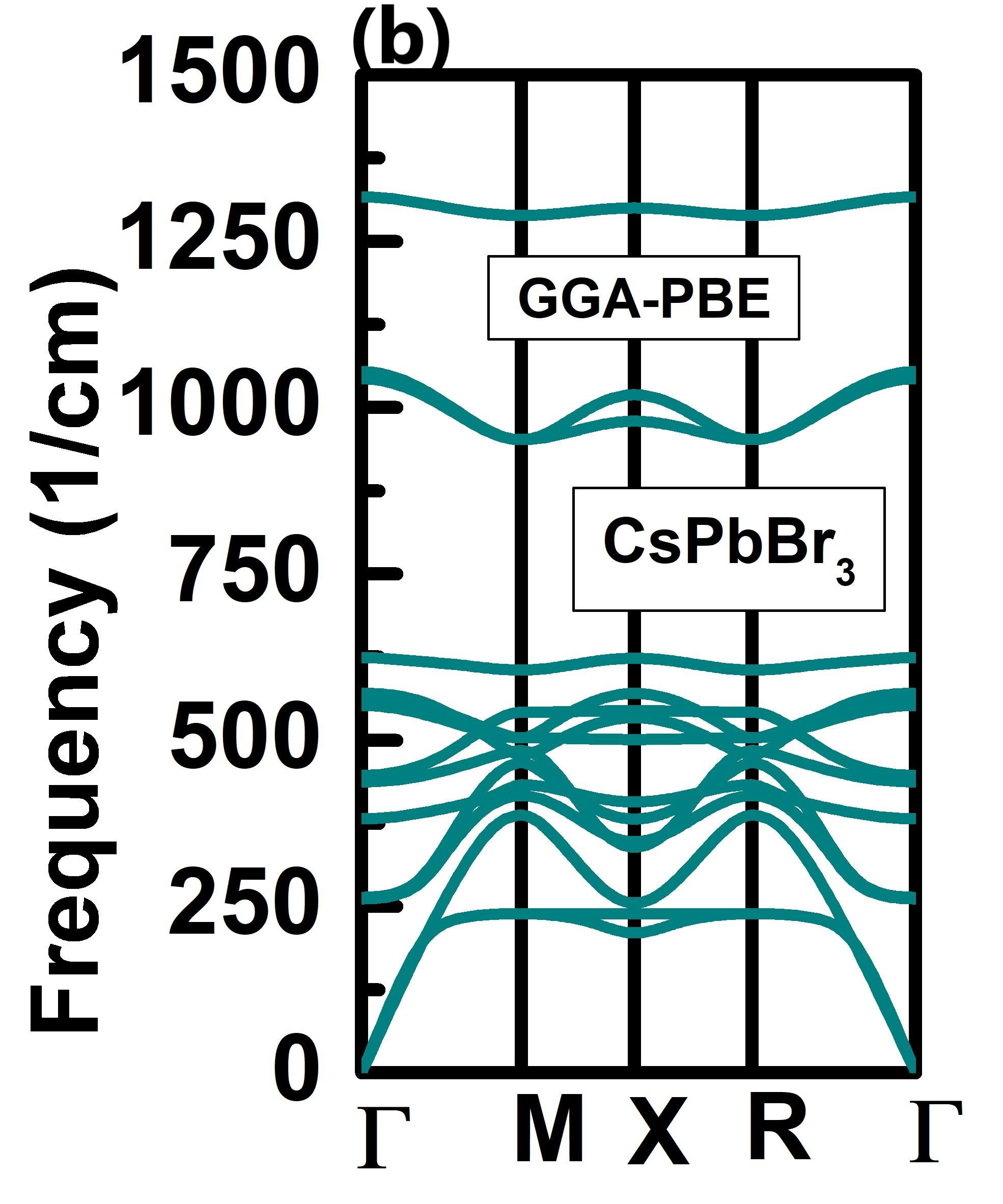 |
| --- | --- |

**Fig. S3.** Phonon dispersion curves of unit cells of (a) CsPbI_3_ monolayer and (b) CsPbBr_3_ monolayer obtained using GGA-PBE parametrization.

**Band structure, TDOS and PDOS of CsPbI_3_ monolayer and CsPbBr_3_ monolayer obtained using GGA-PBE parametrization**

| 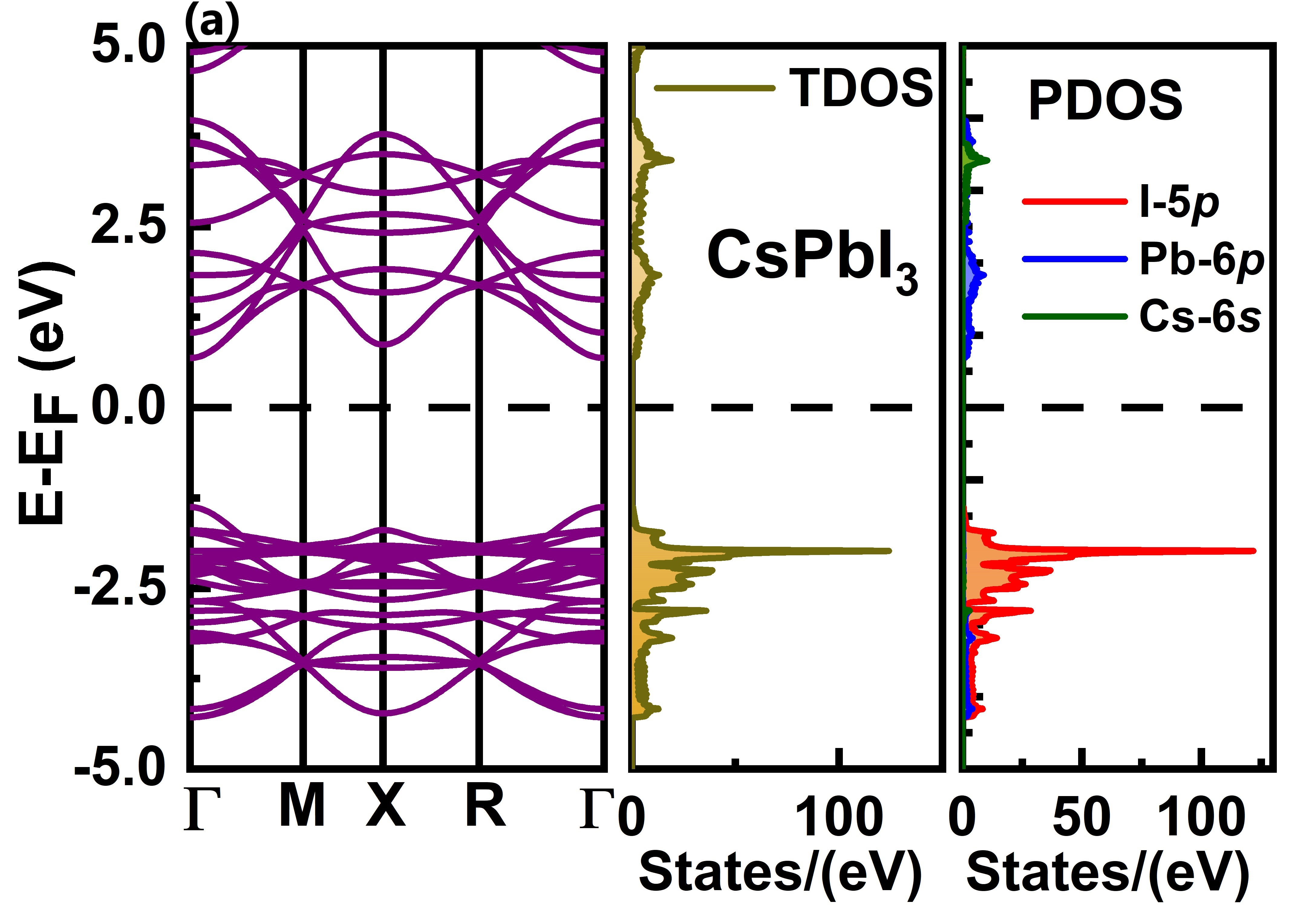 | 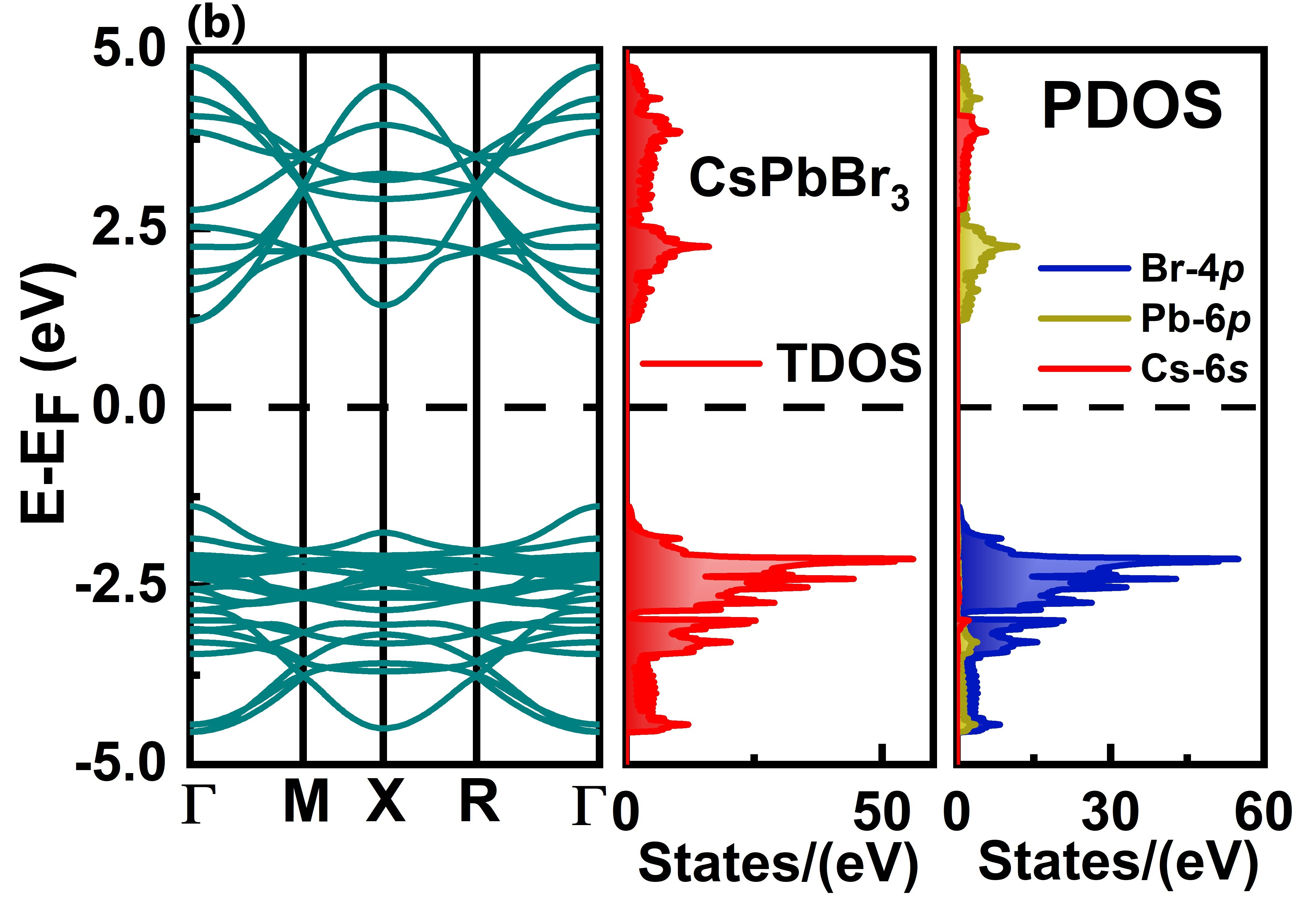 |
| --- | --- |

**Fig. S4.** Band structure, TDOS and PDOS of (a) CsPbI_3_ monolayer and (b) CsPbBr_3_ monolayer obtained within GGA-PBE parametrization. Here electronic properties calculated is of 2 × 2 × 1 monolayer consisting of 20 atoms.

TABLE S3: Bandgaps of CsPbI_3_ monolayer and CsPbBr_3_ monolayer obtained under GGA-PBE parametrization.

| Monolayer | Bandgap (eV) |
| --- | --- |
| CsPbI_3_ | 2.05 |
| CsPbBr_3_ | 2.61 |

**References**

1. Patel, M. J., Gupta, S. K. & Gajjar, P. N. Investigation of Thickness Dependent Efficiency of CsPbX_3_ (X = I, Br) Absorber Layer for Perovskite Solar Cells. *J Phys Chem Solids* **176**, 111264 (2023). <https://doi.org/10.1016/j.jpcs.2023.111264>
